# Supplementary material for: Complexome profiling of the Chlamydomonas psb28 mutant reveals TEF5 as an early PSII assembly factor
Source: Plant Cell. 2025 Mar 17;37(6):koaf055. doi: 10.1093/plcell/koaf055 (PMC12164590; doi:10.1093/plcell/koaf055)
Supplement: koaf055_Supplementary_Data [file koaf055_supplementary_data.zip › psb28_interactive.zip!/plots/intensity/qualityPlots/peptides/MCP14_Cre16.g672650.t1.2.html]

### Cre16.g672650.t1.2

Trivial name: MCP14  
SeqCoverage [median]: 67.30  
Mass: 29.21 kDa  
  
 

| Sequence | MissedCleavage | Mass [Da] | wtIntensity | muIntensity | Score |
| --- | --- | --- | --- | --- | --- |
| AMSLNMGMLASNDQAK | 0 | 1680.76 | 48811.17 | 51895.52 | 199.13 |
| AMSLNMGMLASNDQAKEAIEAAGFPK | 1 | 2694.28 | 4402.67 | 6296.89 | 89.51 |
| AVAGLSAGGIGALVGSPADLTLIR | 0 | 2178.24 | 31464.57 | 0.00 | 228.03 |
| DGVGALYK | 0 | 821.43 | 29012.19 | 148253.17 | 158.36 |
| EAIEAAGFPK | 0 | 1031.53 | 195634.94 | 279649.20 | 231.20 |
| EDGVGGLFR | 0 | 948.47 | 7105.85 | 9828.79 | 104.75 |
| GAAPTVVR | 0 | 769.44 | 225889.21 | 353618.35 | 167.82 |
| GLSAGLLR | 0 | 785.48 | 168206.44 | 356164.05 | 164.89 |
| GSPLAVGAEIVR | 0 | 1167.66 | 98177.08 | 172290.83 | 171.85 |
| GSPLAVGAEIVRK | 1 | 1295.76 | 37668.78 | 106578.71 | 97.86 |
| GVADAFIR | 0 | 847.46 | 159448.11 | 341869.68 | 166.05 |
| IAPHVVFTLVFMDALPK | 0 | 1897.05 | 1374.01 | 0.00 | 91.97 |
| IVKEDGVGGLFR | 1 | 1288.71 | 222023.49 | 672070.62 | 136.01 |
| KDGVGALYK | 1 | 949.52 | 8276.65 | 5050.78 | 96.49 |
| LGIFNMMSEELK | 0 | 1410.69 | 139504.64 | 266039.07 | 248.14 |
| LGIFNMMSEELKAR | 1 | 1637.83 | 78022.69 | 264476.68 | 145.61 |
| MQADATLPVEQR | 0 | 1357.67 | 181080.78 | 188537.14 | 259.35 |
| MQADATLPVEQRR | 1 | 1513.77 | 17418.61 | 75954.49 | 98.53 |
| MTPNPDGTMPYK | 0 | 1350.59 | 2697.22 | 8368.19 | 74.76 |
| NNGQNLPLWQK | 0 | 1310.67 | 1370.32 | 8783.21 | 143.73 |
| NYKGVADAFIR | 1 | 1252.66 | 39347.67 | 168722.91 | 197.73 |
| QATYTTTR | 0 | 940.46 | 78358.10 | 67364.82 | 155.88 |
|  |  |  | 1775295.2 | 3551813.1 |  |
